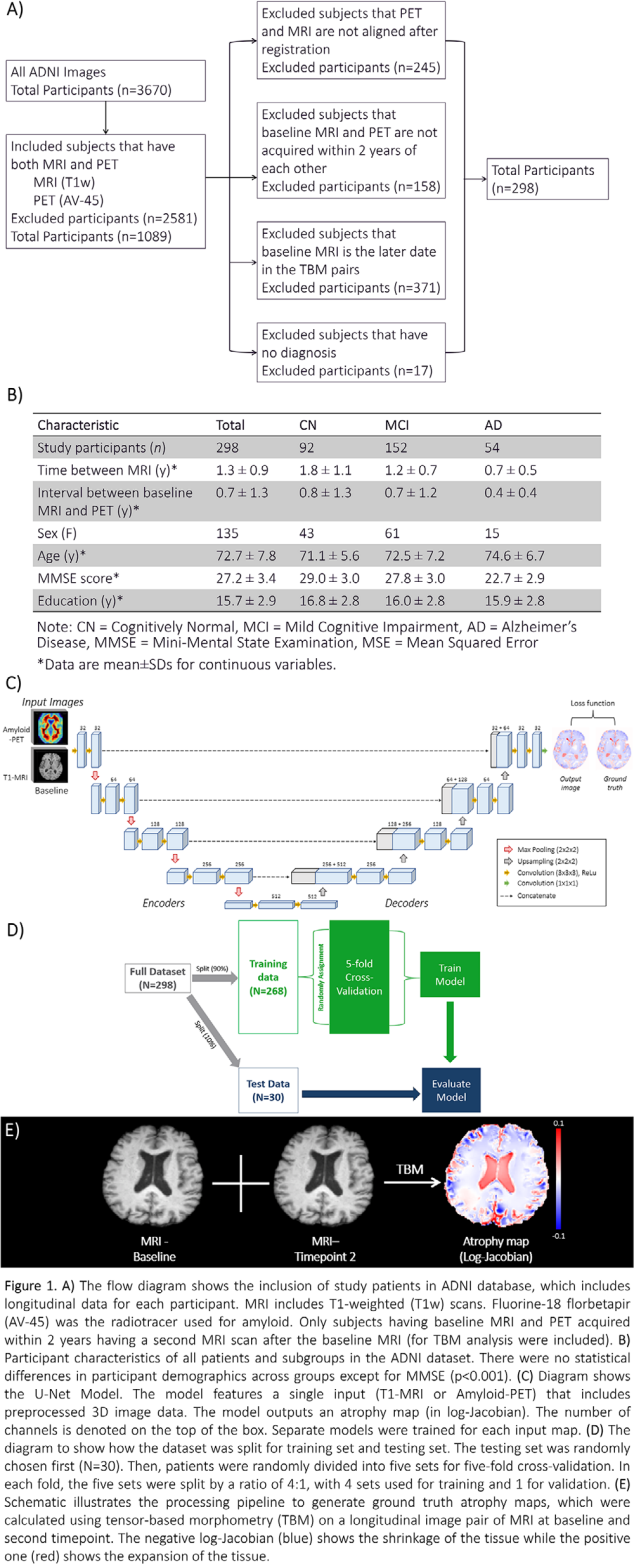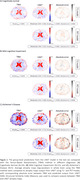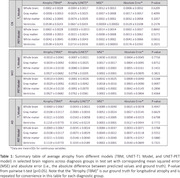# Deep learning‐based estimation of future brain atrophy using baseline MRI and PET

**DOI:** 10.1002/alz.093835

**Published:** 2025-01-09

**Authors:** Linh N. N. Le, Evan Fletcher, Jinyi Qi, Audrey P. Fan

**Affiliations:** ^1^ University of California, Davis, Davis, CA USA; ^2^ University of California Davis, Davis, CA USA

## Abstract

**Background:**

Cortical brain atrophy is an excellent marker of clinical decline and can support future clinical course prediction in cognitive impairment. We used a U‐Net image‐generation deep learning network to predict future cortical atrophy rates in elderly populations, with initial T1‐weighted (T1w‐) MRI or baseline amyloid‐PET serving as inputs to the model.

**Method:**

MRI and PET data were retrospectively collected from Alzheimer’s Disease Imaging Initiative (ADNI) and all participants had two serial T1w‐MRI scans (Figure 1A,B). The ground truth was an atrophy map (in log‐Jacobian values) generated from Tensor Based Morphometry (TBM) of the two longitudinal MRIs (Figure 1E). A U‐Net architecture was used in this study to predict maps of cortical atrophy. We used mean squared error (MSE) for loss function and five‐fold cross‐validation (Figure 1C,D). The testing set was randomly chosen (n = 30). Separate models were trained for T1w‐MRI data as input (T1‐model) and amyloid‐PET as input (PET‐model). The predicted atrophy rate was evaluated across diagnosis groups in the test set using the whole‐brain structural similarity index measure (SSIM) to the TBM ground truth. Each model’s average atrophy at selected brain regions was compared with TBM using paired t‐tests.

**Result:**

Figure 2 shows average predicted atrophy maps and corresponding error for each diagnosis group. In the whole brain, for cognitively normal (CN) and mild‐cognitive‐impaired (MCI) groups, T1 model (with SSIMs of 0.67±0.07 and 0.64±0.06, respectively) slightly outperformed PET model (with SSIMs of 0.70±0.06 and 0.69±0.06, respectively). In selected regions, especially gray matter, we observed a larger rate of atrophy in Alzheimer’s Disease (AD) compared to MCI and in MCI compared to CN groups in both deep learning models, which was consistent with the ground truth. There was no statistical difference in average atrophy predicted by either model and TBM values for all diagnostic groups in all selected regions, with relatively small absolute error and MSE in both models (Table 1).

**Conclusion:**

A U‐Net deep learning model trained using only baseline T1 weighted MRI or amyloid PET data could be used to predict future atrophy rates. Both models predicted cortical volume loss and ventricle expansion that differed between AD, MCI, and CN.